# Endoscopic resection of an Epstein-Barr virus positive inflammatory follicular dendritic cell sarcoma

**DOI:** 10.1055/a-2158-7417

**Published:** 2023-09-15

**Authors:** Ou Chen, Liansong Ye, Liu Que, Bing Hu

**Affiliations:** 1 Department of Gastroenterology and Hepatology, West China Hospital, Sichuan University, Chengdu, China; 2 Department of Gastroenterology, Ya’an People’s Hospital, Ya’an, Sichuan, China; 3 Department of Pathology, Ya’an People’s Hospital, Ya’an, Sichuan, China

A 51-year-old woman was referred to our hospital to check on the 0.5-cm polypoid lesion in the transverse colon that had been detected 2 years previously ( Fig. 1a ). The patient had refused to undergo endoscopic resection of the lesion owing to its small size. She reported no significant discomfort and her medical history was unremarkable. We performed colonoscopy, this time detecting a 3.0-cm pedunculated polypoid lesion in the transverse colon, with congestion and white material on its surface ( Fig. 1b ). A subsequent computed tomography scan showed the lesion to be enhancing ( Fig. 2 ). Endoscopic mucosal resection was performed to completely resect the lesion ( Video 1 ).

**Fig. 1:**
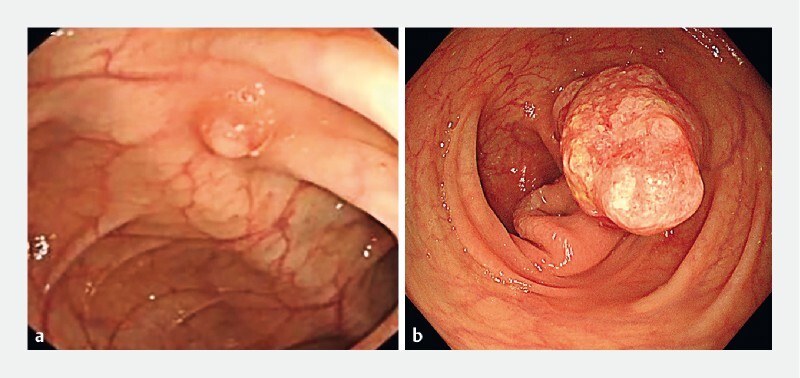
Endoscopic images of the lesion: **a** the 0.5-cm polypoid lesion that was initially detected in the transverse colon 2 years previously, showing the presence of white material on its surface; **b** the 3.0-cm pedunculated polypoid lesion identified on repeat colonoscopy, showing congestion and white material on its surface.

**Fig. 2:**
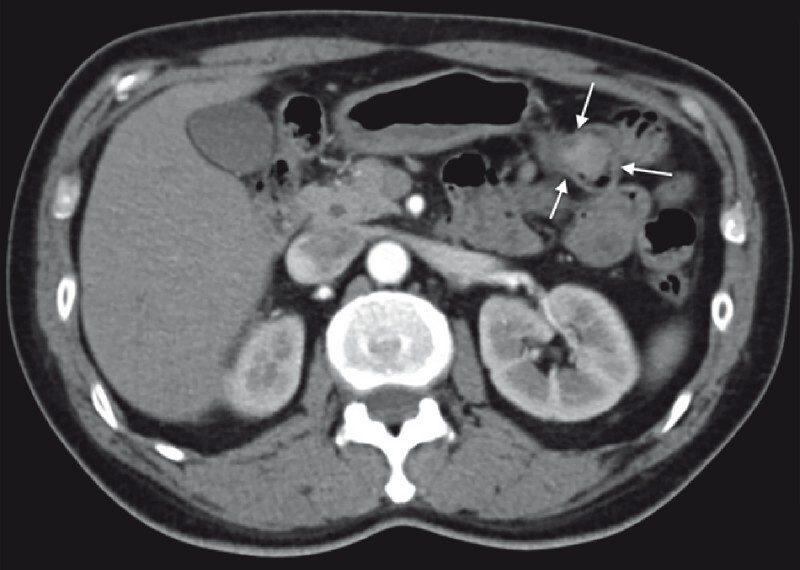
Computed tomography scan showing enhancement of the lesion (arrows).

**Video 1** Endoscopic resection of an Epstein–Barr virus-positive inflammatory follicular dendritic cell sarcoma.


Histopathological examination of the specimen showed a large number of lymphocytes and plasma cells, as well as ovoid to spindle-shaped neoplastic cells. Immunohistochemistry revealed that the tumor cells were positive for CD21, CD23, CD35, CXCL13, D240, and SSTR2, but negative for CK, CD30, CD3, CD20, CD5, CD79, IgD, CD138, and S100. The Ki-67 index was 20 %–30 %. In situ hybridization for EBER was positive ( Fig. 3 ). Finally, the lesion was diagnosed as an Epstein–Barr virus (EBV)-positive inflammatory follicular dendritic cell sarcoma (FDCS) of the colon 1 . Colonoscopy 2 months later showed healing of the wound, and there was no evidence of tumor recurrence ( Fig. 4 ). The patient has remained well during 8 months of follow-up.

**Fig. 3:**
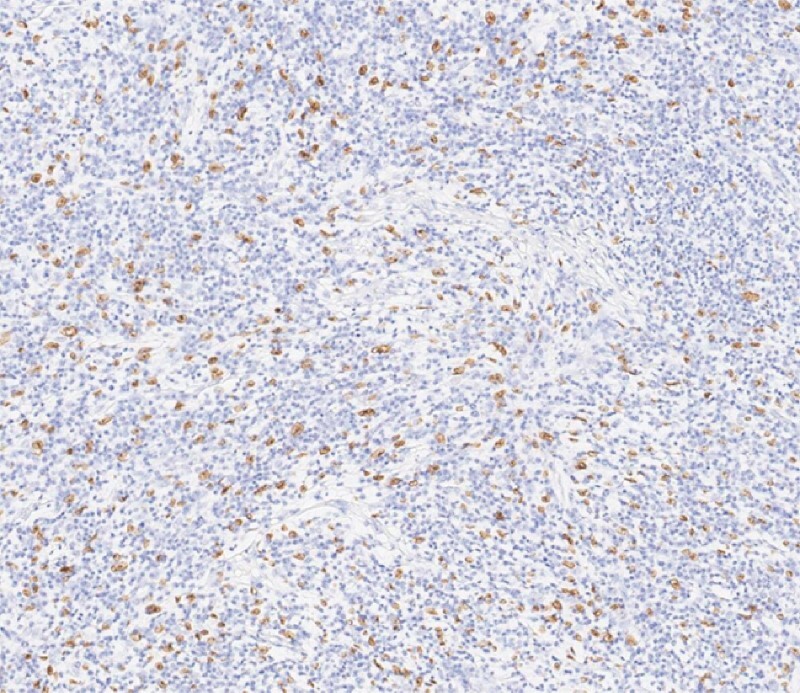
Histopathological appearance of the resected lesion showing positive in situ hybridization for EBER, which along with other immunohistochemical staining was consistent with the diagnosis of an Epstein–Barr virus-positive inflammatory follicular dendritic cell sarcoma of the colon.

**Fig. 4:**
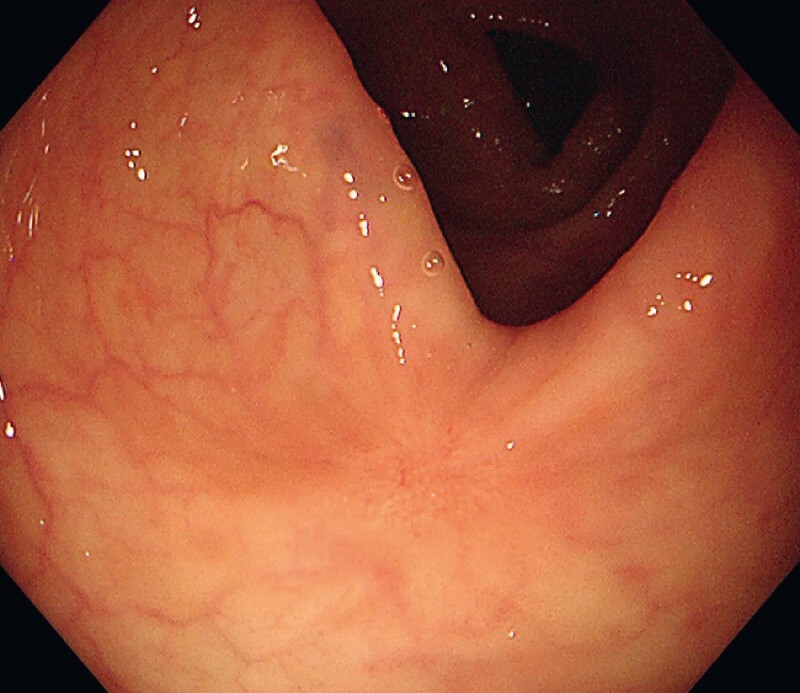
Colonoscopic appearance 2 months after resection showing healing of the wound and no evidence of tumor recurrence.

EBV-positive inflammatory FDCS is a rare type of neoplasm, which can be observed in the spleen, liver, and other organs, but rarely in the colon 2 3 . Extranodal FDCS is usually indolent, 2 but in this case, the tumor grew quickly during a 2-year interval, which could have caused intestinal obstruction if the lesion had been neglected. Compared with a routine polyp, the main characteristic of this colonic EBV-positive inflammatory FDCS was the presence of white material on its surface, even when the lesion was relatively small. Targeted biopsy should be performed for such lesions during screening colonoscopy.

Endoscopy_UCTN_Code_CCL_1AD_2AZ

## Notes

Endoscopy E-Videos

https://eref.thieme.de/e-videos
*E-Videos* is an open access online section of the journal *Endoscopy* , reporting on interesting cases and new techniques in gastroenterological endoscopy. All papers include a high-quality video and are published with a Creative Commons CC-BY license. Endoscopy E-Videos qualify for HINARI discounts and waivers and eligibility is automatically checked during the submission process. We grant 100% waivers to articles whose corresponding authors are based in Group A countries and 50% waivers to those who are based in Group B countries as classified by Research4Life (see: https://www.research4life.org/access/eligibility/ ). This section has its own submission website at https://mc.manuscriptcentral.com/e-videos
